# DNA damage response inhibition at dysfunctional telomeres by modulation of telomeric DNA damage response RNAs

**DOI:** 10.1038/ncomms13980

**Published:** 2017-02-27

**Authors:** Francesca Rossiello, Julio Aguado, Sara Sepe, Fabio Iannelli, Quan Nguyen, Sethuramasundaram Pitchiaya, Piero Carninci, Fabrizio d’Adda di Fagagna

**Affiliations:** 1 IFOM, the FIRC Institute of Molecular Oncology, Via Adamello 16, 20139 Milan, Italy; 2 RIKEN Center for Life Science Technologies, Division of Genomic Technologies, Yokohama, Kanagawa 230‐0045, Japan; 3 Michigan Center for Translational Pathology, University of Michigan Cancer Center, MI 48109, Ann Arbor, USA; 4 Istituto di Genetica Molecolare, Consiglio Nazionale delle Ricerche (IGM-CNR), Via Abbiategrasso 207, 27100 Pavia, Italy

## Abstract

The DNA damage response (DDR) is a set of cellular events that follows the generation of DNA damage. Recently, site-specific small non-coding RNAs, also termed DNA damage response RNAs (DDRNAs), have been shown to play a role in DDR signalling and DNA repair. Dysfunctional telomeres activate DDR in ageing, cancer and an increasing number of identified pathological conditions. Here we show that, in mammals, telomere dysfunction induces the transcription of telomeric DDRNAs (tDDRNAs) and their longer precursors from both DNA strands. DDR activation and maintenance at telomeres depend on the biogenesis and functions of tDDRNAs. Their functional inhibition by sequence-specific antisense oligonucleotides allows the unprecedented telomere-specific DDR inactivation in cultured cells and *in vivo* in mouse tissues. In summary, these results demonstrate that tDDRNAs are induced at dysfunctional telomeres and are necessary for DDR activation and they validate the viability of locus-specific DDR inhibition by targeting DDRNAs.

## Abstract (web summary)

The DNA damage response (DDR) involves site-specific small non-coding RNAs. Here the authors show that telomere dysfunction induces transcription of telomeric DNA damage response RNAs that are necessary for DDR activation, which can be specifically muted by antisense inhibitory oligonucleotides.

The DNA damage response (DDR) is a coordinated signalling network involving a large number of interacting proteins controlled by post-translational modifications and includes signalling, cell–cycle checkpoint enforcement and DNA repair^1^,^2^. Following DNA double-strand break (DSB) generation, the apical DDR kinase ataxia telangiectasia mutated (ATM) undergoes activation and phosphorylates the histone H2AX at serine 139 (named γH2AX); this event is necessary for the recruitment of DDR proteins to sites of DNA damage, including ATM phosphorylated at serine 1,981 (pATM) itself and p53-binding protein 1 (53BP1). Recently, a class of small non-coding RNAs (ncRNAs), termed DNA damage response RNAs (DDRNAs), has been shown to be generated on transcription of the damaged locus following DSB induction and then processed by the endoribonucleases DICER and DROSHA^3^,^4^,^5^. DDRNAs are dispensable for the direct recognition of DNA damage and γH2AX phosphorylation, but necessary for the secondary recruitment of DDR proteins to DSBs to form the so-called DDR foci^6^. A similar set of small ncRNAs, named damage-induced RNAs, has been shown to be involved in DNA repair by homologous recombination (HR) in plants and human cells^7^,^8^, and by non-homologous end-joining (NHEJ) in plants^9^.

Telomeres are the nucleoprotein complexes located at the tips of eukaryotic chromosomes, composed of repetitive DNA (TTAGGG in vertebrates), and coated by a set of proteins collectively known as the shelterin complex^10^. Dysfunctional telomeres resemble DSBs and they have been observed during ageing, cancer and a number of medical conditions^11^,^12^,^13^,^14^,^15^,^16^,^17^. Apart from telomeric repeat-containing RNA (TERRA), a non-coding UUAGGG-rich transcript starting from promoters located in the subtelomeric region^18^,^19^, in mammals no other transcripts at telomeres have been characterized so far.

Here we provide evidence that both strands of deprotected telomeres are transcribed to generate telomeric DDRNAs (tDDRNAs) and their precursors, whose inhibition leads to a reduction in the DDR activation at dysfunctional telomeres in living cells and *in vivo*.

## Results

### Telomere deprotection triggers both strands’ transcription

**Figure 1:**
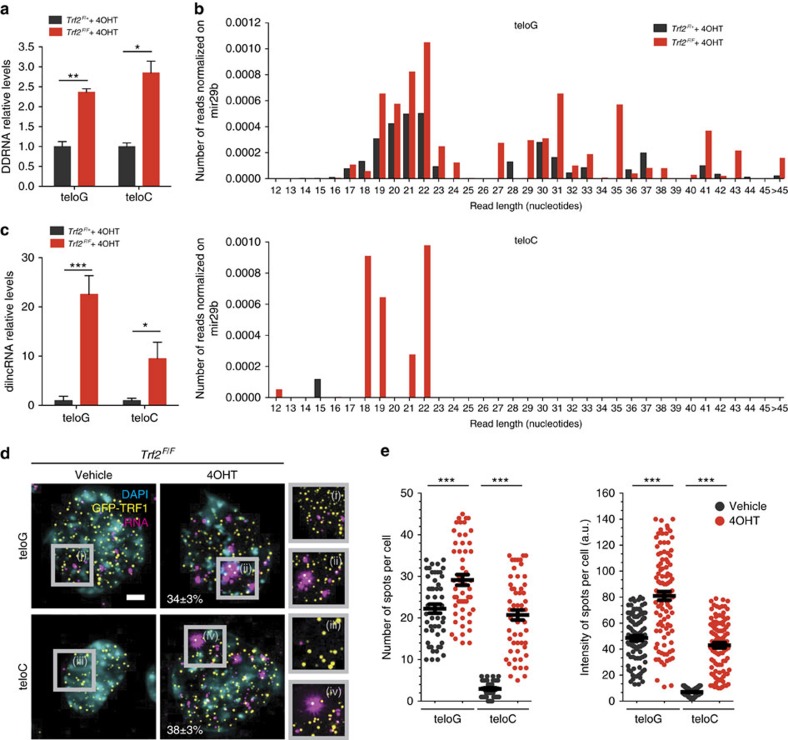
Deprotection of telomeres leads to enhanced transcription of both telomere DNA strands. (**a**) Total cell RNA was isolated from MEFs of the indicated genotype and treated with 4-hydroxytamoxifen (4OHT). Gel-extracted small RNA fraction (<40 nucleotides) was used for miScript PCR amplification to specifically detect DDRNAs. Error bars represent the s.e.m. *n*=3 independent experiments. *P<0.05, **P<0.01, Student’s *t*-test. (**b**) Small RNA (<200 nucleotides) fractions were isolated from 4OHT-treated MEFs of the indicated genotype, enriched for species with telomeric sequences using a telomeric bait, and sequenced. Histograms show for each reported read length the number of telomeric reads, either G rich or C rich, normalized on mir29b reads. For both teloG and teloC, MEFs *Trf2*^*F/F*^ have a significantly higher proportion of small RNA with respect to mir29b reads than MEFs *Trf2*^*F/+*^. *P*<0.001, Fisher’s exact test. (**c**) Total cell RNA was isolated from MEFs of the indicated genotype and used for strand-specific RT–qPCR to detect telomeric dilncRNAs. Error bars represent the s.e.m. *n*=3 independent experiments. *P<0.05; ***P<0.001, Student’s *t*-test. (**d**,**e**) MEFs *Trf2*^*F/F*^-expressing GFP-TRF1 were treated with vehicle or 4OHT and analysed 48 h later. (**d**) Representative images of teloG or teloC dilncRNA transcripts by smFISH. Zoomed in view of the boxed regions (12.5 × 12.5 μm^2^), with GFP-TRF1 and FISH signals, is shown on the right. The indicated numbers show the percentage of RNA signals co-localizing with GFP-TRF1±s.e.m. Scale bar, 5 μm (**e**) Quantification of data presented in **d**, showing the number of spots per cell and the relative intensity (a.u.=arbitrary units). Lines depict the mean±s.e.m. *n*=2 independent experiments; at least 50 cells per sample have been analysed; ***P<0.001.

To explore the potential generation of DDRNAs and their role at dysfunctional telomeres, we used *Trf2* conditional knockout mouse embryonic fibroblasts (*Trf2*^*F/*+^ and *Trf2*^*F/F*^ mouse embryonic fibroblasts (MEFs)) carrying a Cre recombinase (*Rosa26-CreERT2*) inducible by 4-hydroxytamoxifen (4OHT)^20^. TRF2 is a component of the shelterin complex and prevents telomeric DNA from being recognized as a DSB^10^. Recombination by Cre promptly and robustly induced telomere deprotection and DDR activation at telomeres in the *Trf2*^*F/F*^ cell line only (Supplementary Fig. 1a; ref. 20). DDRNAs are small double-stranded RNAs (dsRNAs) transcribed at DSBs and carry the sequence of the damaged genomic locus^21^. To study tDDRNAs we employed miScript, a quantitative PCR with reverse transcription (RT–qPCR)-based method^22^ developed to detect and quantify small RNAs, and designed primers to selectively amplify either strands of potential tDDRNAs generated at deprotected telomeres: teloC (C-rich strand of tDDRNA) and teloG (G-rich strand of tDDRNA; Supplementary Fig. 1b). Using a gel-extracted fraction of RNAs shorter than 40 nucleotides—thus, minimizing the contribution of much longer telomeric transcripts, including TERRA^18^,^19^,^23^—we observed an induction of both teloC and teloG RNAs (Fig. 1a), thus with the potential to form *in vivo* double-stranded tDDRNA molecules. The signals detected were not due to genomic DNA contamination, since very low signal was detectable in the absence of reverse transcription (Supplementary Fig. 1c). To extend these observations beyond mouse cells or TRF2 genetic ablation only, we used T19 cells, a human HT1080 fibrosarcoma cell line derivative, expressing a doxycycline-induced FLAG-tagged dominant negative form of TRF2 (*TRF2 ΔBΔM*), whose expression leads to telomere deprotection and DDR activation (Supplementary Fig. 1d,e; ref. 24). We observed that also in this cell system, tDDRNA levels increased following telomere deprotection (Supplementary Fig. 1f). This indicates that tDDRNA induction mechanisms are conserved among species.

To further characterize the length and sequence of tDDRNAs, we devised and employed an innovative method for target enrichment of RNA, based on in solution capture of low-abundance small RNA species followed by next-generation sequencing, developed in our laboratories (Nguyen *et al*., in preparation). By this approach, we observed that telomere deprotection induced the accumulation of small RNA species generated from the transcription of both telomere strands, including the expected DDRNA size range products (Fig. 1b; Supplementary Fig. 1g). Interestingly, 20–23 nucleotide RNAs displayed a base bias at both 5′- and 3′- ends significantly different from the telomeric locus (Supplementary Fig. 2a), suggesting a regulated processing.

We next sought evidence for longer RNA species—we named them damage-induced long ncRNAs (dilncRNAs)—potential precursors of tDDRNAs. For this purpose, we performed a strand-specific fixed-length RT–qPCR^25^ that allows the quantitative amplification of repetitive sequences, such as telomeric repeats, but not of RNA molecules shorter than 40 nucleotides such as DDRNAs (Supplementary Fig. 2b). In cells with deprotected telomeres, both teloC and teloG long transcripts were 10- to 20-fold more abundant than in control cells (Fig. 1c), indicating that telomere deprotection strongly induces telomere transcription on both strands. Although PCR amplification of UUAGGG repeat-containing RNA may also detect the well-characterized telomeric transcript TERRA^18^,^19^, C-rich telomere repeat-containing RNA has not been characterized in mammals. To generate additional evidence of the telomere transcription induced on telomere deprotection by an independent approach, and to dissect the sub-cellular localization of telomeric dilncRNAs, we employed single-molecule fluorescence *in situ* hybridization (smFISH)^26^. To visualize telomeres in *Trf2*^*F/F*^ MEFs, we stably expressed a green fluorescent protein (GFP)-tagged version of the telomeric protein TRF1, which co-localized with γH2AX foci on telomere deprotection (Supplementary Fig. 2c,d). By using strand-specific DNA probes, nuclear focal signals of RNA transcripts with telomeric sequence were detected. Strikingly, on telomere deprotection, we observed a marked increase in the number and the intensity of such nuclear signals for both G- and C-rich telomeric RNA, which often co-localized with GFP-TRF1 signals, and much less with centromeres used as a negative control (Fig. 1d,e; Supplementary Fig. 2e). In control cells expressing TRF2, a weaker signal was detected only by the probe against teloG, which likely detects also TERRA transcripts, while none was detected by probing for the complementary strand. The observed signals were specific for RNA, since they were lost when RNase A was added before hybridization (Supplementary Fig. 2f). This result further strengthens the evidence of transcriptional induction of both DNA strands at deprotected telomeres.

### DDR at deprotected telomeres is DICER and DROSHA dependent

**Figure 2:**
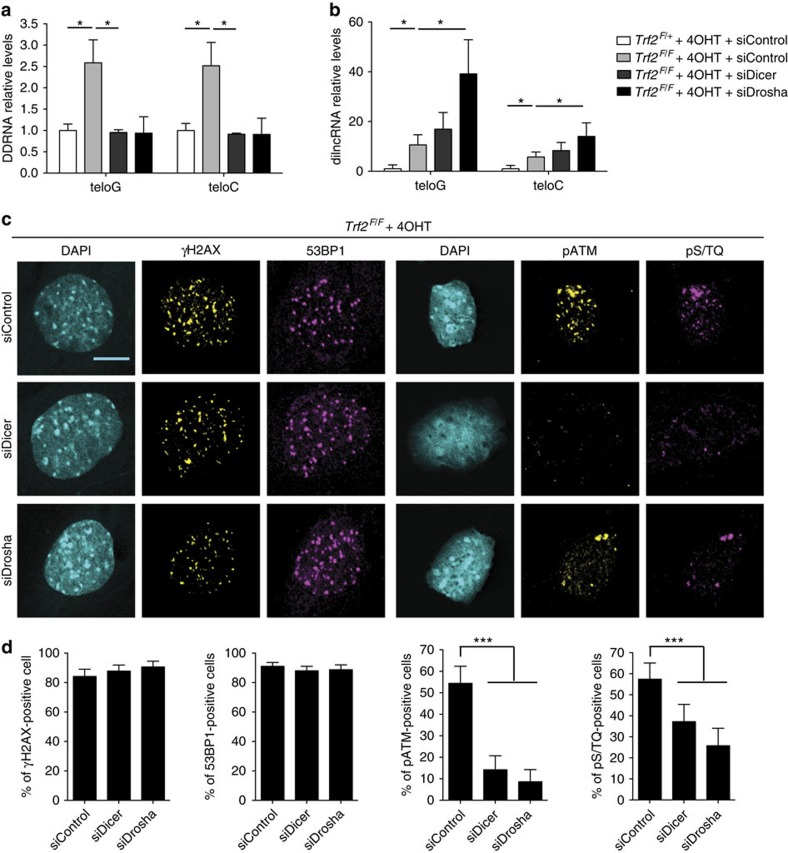
DICER and DROSHA are involved in dilncRNA processing and DDRNA generation and necessary for full DDR activation at deprotected telomeres. (**a**–**b**) Total cell RNA was isolated from MEFs of the indicated genotype that were previously treated with 4-hydroxytamoxifen (4OHT) and transfected with the indicated siRNA. (**a**) Gel-extracted small RNA fraction (<40 nucleotides) was used for miScript PCR amplification to specifically detect DDRNAs. Error bars represent the s.e.m. *n*=3 independent experiments. *P<0.05, Student’s *t*-test. (**b**) Total RNA was used for strand-specific RT–qPCR. Error bars represent the s.e.m. *n*=3 independent experiments. *P<0.05, Student’s *t*-test. (**c**) MEFs *Trf2*^*F/F*^ were treated with 4OHT, transfected with the indicated siRNA and stained for the indicated DDR markers. Scale bar, 10 μm. (**d**) Quantification of data presented in **c**. Bar graphs show the percentage of DDR-positive cells±95% confidence interval *n*=3 independent experiments; at least 150 cells per sample have been analysed; ***P<0.001, *χ*^2^-test.

DICER and DROSHA have been shown to be involved in DDRNA biogenesis on DSB generation^21^. We therefore investigated their role in the processing of telomeric transcripts, and consequently in DDR activation at dysfunctional telomeres, by individually knocking down each of them in *Trf2*^*F/F*^ MEFs (Supplementary Fig. 2g). We observed that silencing of DICER or DROSHA fully abolished tDDRNA induction following telomere deprotection (Fig. 2a). Conversely, in telomere-deprotected cells, telomeric dilncRNAs increased significantly in *Drosha* knocked-down cells (Fig. 2b). This accumulation is consistent with dilncRNAs being DDRNA precursors, which are then processed by DROSHA—an increase on *Dicer* knockdown could not be appreciated likely because *Dicer* knockdown leads to the accumulation of RNA intermediates too short to be detected by qPCR in this experimental setting.

Next, we aimed to study the impact of the DDRNA biogenesis inhibition on DDR activation through *Dicer* or *Drosha* knockdown. In MEFs exposed to ionizing radiation (IR), as reported in other cell lines^21^, *Dicer* and *Drosha* knockdown impaired 53BP1 and pATM foci formation, while γH2AX foci remained unchanged (Supplementary Fig. 2h,i). This was not due to altered 53BP1 or ATM protein levels (Supplementary Fig. 3a,b), consistent with the reported unaltered levels of chromosomal integrity genes and telomere maintenance factors observed in conditional knockout mouse models for *Dicer* or *Dgcr8*, a *Drosha* cofactor^27^.

We then tested whether DICER and DROSHA have a role in DDR activation at dysfunctional telomeres. As expected, DICER or DROSHA loss did not affect γH2AX foci at deprotected telomeres (Fig. 2c,d). When we studied the activation of the DDR apical kinase ATM, we observed that the fraction of cells containing pATM foci, as well as foci of proteins phosphorylated by PI3-like kinases, including ATM on their consensus target sequence (pS/TQ), were strongly reduced on *Dicer* and *Drosha* knockdown (Fig. 2c,d). 53BP1 foci formation was not detectably affected on telomere deprotection in *Dicer* and *Drosha* knocked-down cells (Fig. 2c,d); this is consistent with the notion that 53BP1 recruitment to DDR foci is controlled by redundant mechanisms^28^ and is most dependent on DICER and DROSHA only at early time points (10 min) following DNA damage generation^21^, while the genetic deletion of TRF2 does not allow such early time points studies. Unbiased identification and quantification of DDR foci by an automatic imaging software^29^ confirmed that in both DICER and DROSHA-depleted cells, the number, as well as the intensity, of pATM and pS/TQ foci per cell were reduced, whereas γH2AX and 53BP1 foci were unaffected (Supplementary Fig. 3c). Furthermore, in human T19 cells, both *Dicer* and *Drosha* knockdown reduced the recruitment of pATM and ATM kinase targets pS/TQ, to deprotected telomeres, but not γH2AX foci formation (Supplementary Fig. 3d,e). Therefore, in both mouse and human cells the stable recruitment of activated ATM and its phosphorylated targets to deprotected telomeres requires DICER and DROSHA.

DICER-dependent small ncRNAs generated at sites of DNA damage, damage-induced RNAs, have been proposed to be necessary for efficient repair by HR in *Arabidopsis thaliana* and human cells^7^,^8^. While DICER seems dispensable for NHEJ DNA repair in a human cell-based reporter system^8^, Dicer-like 3 has been involved in NHEJ in plants^9^. More recently, error-free DSBs repair by NHEJ have been shown to involve RNA transcripts^30^, while transient DNA–RNA hybrids seem to be necessary for HR in fission yeast^31^. Chromosomal fusions on TRF2 depletion are DNA repair events mediated by NHEJ^32^. We therefore tested whether DICER or DROSHA have a role in NHEJ events at dysfunctional telomeres. Following telomere deprotection, siControl-transfected MEFs showed massive chromosomal fusions, as expected (Supplementary Fig. 4a; ref. 33). Instead, *Dicer* and *Drosha* knockdown reduced the number of telomere fusions, reaching statistical difference in the case of DROSHA inactivation (Supplementary Fig. 4a,b). These results indicate that DICER and DROSHA, and likely their DDRNAs products, are involved in NHEJ DNA repair at dysfunctional telomeres in mammalian cells.

### DDR at deprotected telomeres is mediated by tDDRNAs

**Figure 3:**
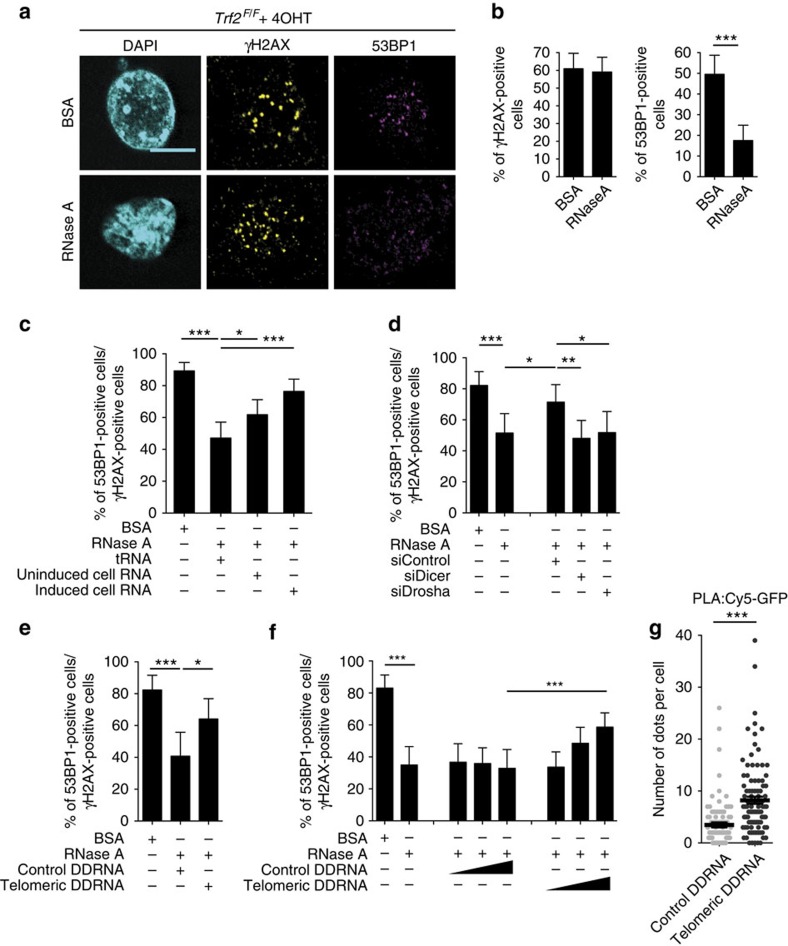
DDR foci formation at deprotected telomeres is DDRNA dependent. (**a**) MEFs *Trf2*^*F/F*^ were treated with 4-hydroxytamoxifen (4OHT), permeabilised, treated with RNase A, or BSA as control, and stained for the indicated DDR markers. Scale bar, 20 μm. (**b**) Quantification of data presented in **a**. Bar graphs show the percentage of DDR-positive cells±95% confidence interval (CI); *n*=3 independent experiments; at least 100 cells per sample have been analysed; ***P<0.001, *χ*^2^-test. (**c**–**f**) After RNase A treatment, cells were incubated with: (**c**) cell RNA from MEFs *Trf2*^*F/F*^ with normal (uninduced) or deprotected (induced) telomeres, or tRNA as a control; (**d**) cell RNA from MEFs *Trf2*^*F/F*^ treated with 4OHT and transfected with the indicated siRNA; (**e**) small double-stranded RNAs generated by recombinant DICER; (**f**) synthetic double-stranded RNAs. Bar graphs show the percentage of 53BP1-positive cells normalized on γH2AX-positive cells±95% CI *n*=3 independent experiments; at least 100 cells per sample have been analysed; *P<0.05, **P<0.01, ***P<0.001, *χ*^2^-test. (**g**) MEFs *Trf2*^*F/F*^-expressing GFP-TRF1 were permeabilized and incubated with the indicated Alexa Fluor 647-conjugated double-stranded RNAs. The graph shows the mean number of dots per cell±s.e.m., detected by proximity ligation assay (PLA), using an anti-GFP antibody, recognizing the telomeric protein TRF1 and an anti-Cy5 antibody, recognizing the Alexa Fluor 647 antigen; *n*=2 independent experiments; at least 80 cells per sample have been analysed; ***P<0.001, Student’s *t*-test.

Thus far, the demonstrated effects of DICER and DROSHA on DDR activation at deprotected telomeres do not exclude the possibility that such effects may be indirect, meaning mediated by the role of DICER and DROSHA in microRNAs (miRNAs) biogenesis and gene expression. To address this issue, we used an experimental set-up, in which we previously demonstrated^21^ that the impact on DDR activation of DICER- and DROSHA-generated DDRNAs can be studied independently from the canonical translational effects of miRNAs. We thus used a mild detergent to transiently permeabilize cell membranes of *Trf2*^*F/F*^ MEFs, in which DDR was triggered by IR or by *Trf2* genetic ablation and treated them with RNase A, or bovine serum albumin (BSA) as control. Both IR- and telomere deprotection-induced 53BP1 foci were sensitive to RNase A (Fig. 3a,b; Supplementary Fig. 4c,d), despite unaltered 53BP1 protein levels (Supplementary Fig. 4e,f), while γH2AX foci were not affected. Thus, also at deprotected telomeres RNA is necessary for DDR foci maintenance. To test if DDR foci can reform in an RNA-dependent manner, permeabilised RNase A-treated, and telomere-deprotected, MEFs were incubated with RNA extracted from MEFs with either normal (uninduced) or deprotected (induced) telomeres, or yeast transfer RNA (tRNA) as control. We observed that only RNA from induced MEFs allowed the robust reformation of 53BP1 foci in RNase A-treated cells (Fig. 3c). We then characterized the RNA species required for DDR foci reformation and observed that RNA extracted from induced cells knocked down for *Dicer* or *Drosha* was unable to restore 53BP1 foci (Fig. 3d). This indicates that the RNA species responsible for DDR foci maintenance at dysfunctional telomeres are DICER and DROSHA products. To further demonstrate that telomeric DICER products are indeed the RNA molecules necessary for DDR activation at telomeres and are sufficient to allow telomeric DDR foci reformation following RNase A treatment, we generated small double-stranded tDDRNAs by incubating a long double-stranded telomeric, or control, RNA substrate with purified recombinant DICER (Supplementary Fig. 4g). We then added these *in vitro*-generated telomeric and control DDRNAs to telomere-deprotected, RNase A-treated MEFs. We observed that only telomeric, but not control, DICER products could restore 53BP1 foci formation (Fig. 3e). Importantly, we can exclude that this effect was mediated by miRNA-like translational inhibition by DICER products, since messenger RNAs, the potential targets, were degraded by RNase A treatment and the effects were observed within minutes from RNA addition. To further prove the crucial dependency of DDR signalling at telomeres on tDDRNAs, we chemically synthetized 21-nucleotide long dsRNAs with a telomeric or a control sequence and tested their activity in RNase A-treated *Trf2*-deleted MEFs. We observed that the addition of synthetic tDDRNAs was sufficient to allow the reformation of robust DDR signalling events, as indicated by the reformation of 53BP1 foci, while DDRNAs with a control sequence had no effect (Fig. 3f). Consistent with a role of tDDRNAs in 53BP1 foci reformation at dysfunctional telomeres, tDDRNAs localized to dysfunctional telomeres, as measured by proximity ligation assay (PLA) between GFP-tagged TRF1 and Alexa 647-conjugated to tDDRNAs, or control DDRNAs that localized to telomeres much less (Fig. 3g; Supplementary Fig. 4h). In summary, these results demonstrate that tDDRNAs have a crucial role in DDR activation at dysfunctional telomeres.

### Antisense oligonucleotides allow DDR inhibition at telomeres

**Figure 4:**
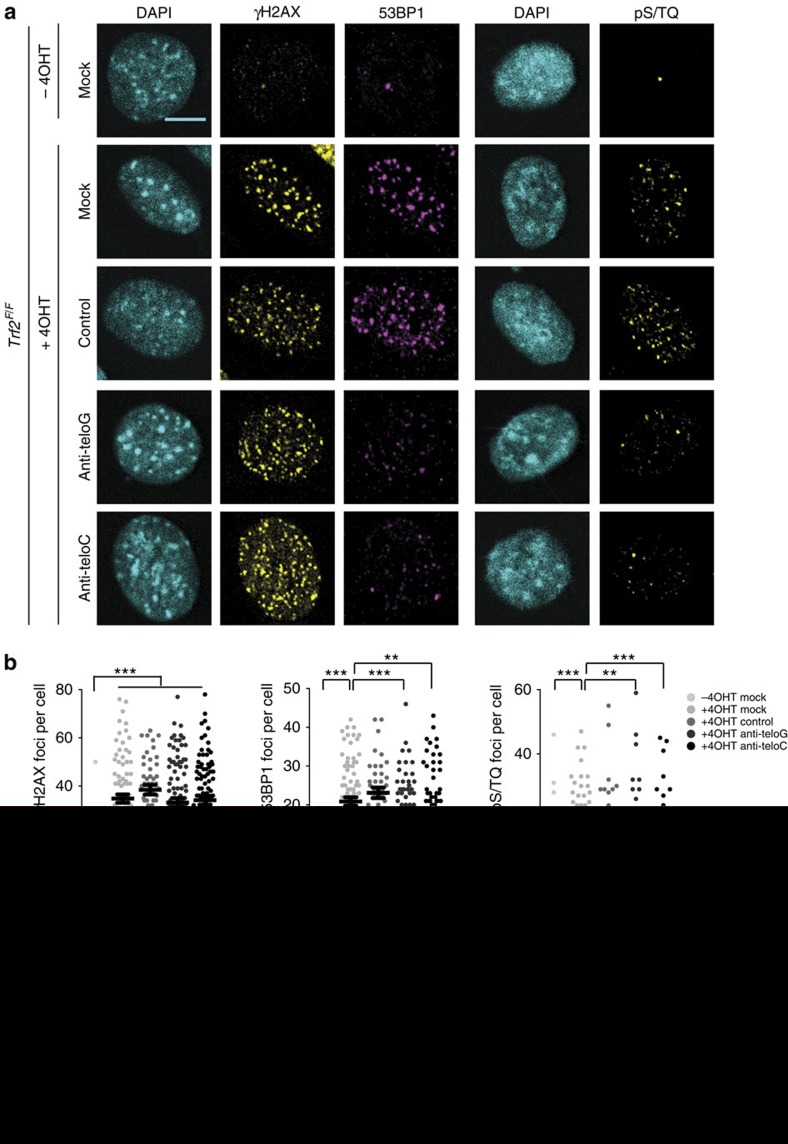
Antisense oligonucleotides against tDDRNAs inhibit DDR activation at deprotected telomeres. (**a**) MEFs *Trf2*^*F/F*^ were treated, or not, with 4-hydroxytamoxifen (4OHT), transfected with the indicated ASO, and stained for the indicated DDR markers. Scale bar, 10 μm (**b**) Quantification of data presented in **a**. Dot plots show the number or intensity of DDR foci per cell (a.u.=arbitrary units). Lines depict the mean±s.e.m. *n*=3 independent experiments; at least 50 cells per sample have been analysed; **P<0.01, ***P<0.001, one-way analysis of variance with Sidak corrections for multiple comparisons.

The results described so far consistently support the notion of a crucial role of tDDRNAs in DDR signalling at deprotected telomeres. On the grounds of the above conclusions, we next tested whether DDR at telomeres could be affected by the direct inhibition of tDDRNA functions. Inhibitory antisense oligonucleotides (ASOs) are widely used to block the functions of RNA molecules, including ncRNAs and small RNAs such as miRNAs^34^ by steric hindrance. We therefore designed ASOs complementary to either tDDRNAs (anti-teloG and anti-teloC) or an ASO with an unrelated control sequence. We observed no obvious increase in γH2AX, 53BP1, pATM and pS/TQ foci in wild-type MEFs transfected with any of the ASOs used, ruling out the possibility that ASOs could compete with telomeres for the binding of telomeric proteins (Supplementary Fig. 5a,b). ASOs were then transfected in MEFs before telomere deprotection, or exposure to IR as a source of non sequence-specific DNA damage, and DDR activation was monitored. ASO treatment did not have any impact on IR-induced DDR foci (Supplementary Fig. 6a). Differently, both anti-teloG and anti-teloC, but not the control ASO, reduced both the number and the intensity of 53BP1 and pS/TQ foci at deprotected telomeres (Fig. 4a,b). Similar to *Dicer* and *Drosha* knockdown, and RNase A treatment, no effect on γH2AX foci was observed.

To test the direct role of tDDRNAs on chromosomal fusions following TRF2 depletion, we inhibited them by ASOs in cultured MEFs and monitored fusion events. We observed a significant reduction in the frequency of chromosomal fusions in cells treated with both anti-teloG and anti-teloC ASOs, but not with the control ASO (Supplementary Fig. 6b).

These results demonstrate, for the first time, that DDR activation and NHEJ events at dysfunctional telomeres can be modulated in living cells by ASOs.

### Telomeric DDR inhibition *in vivo*

**Figure 5:**
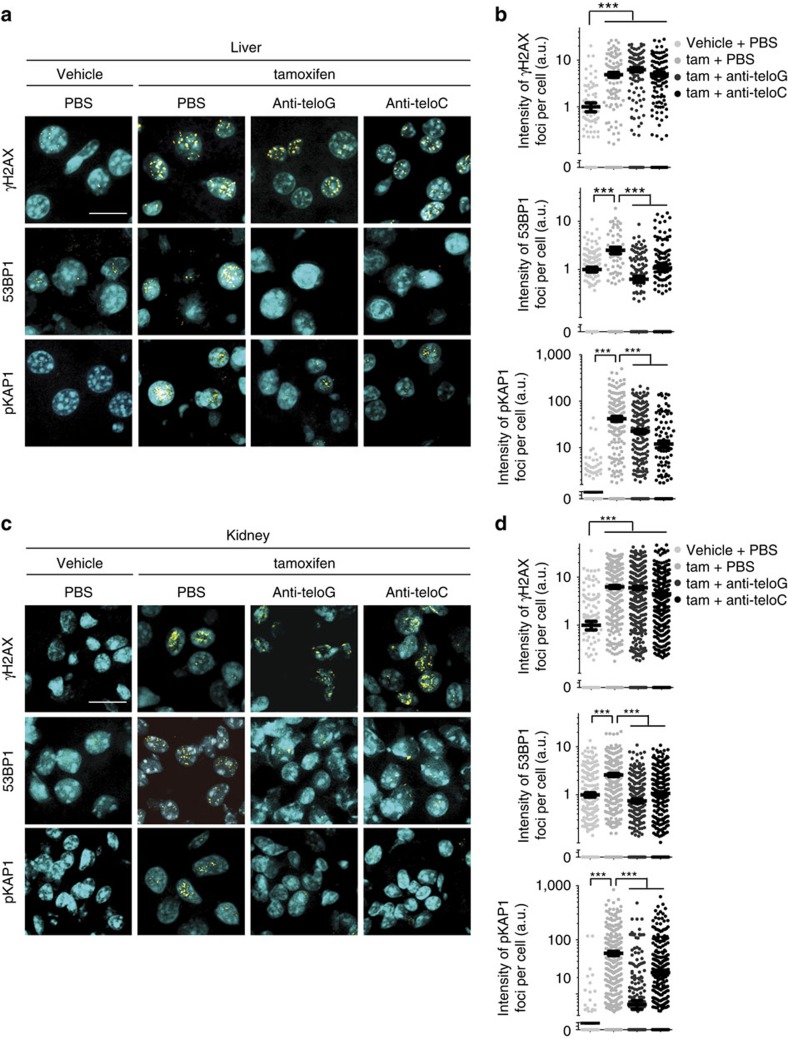
Antisense oligonucleotides against tDDRNAs inhibit DDR activation *in vivo* in mice with deprotected telomeres. (**a**–**d**) Mice were injected with vehicle or tamoxifen (tam) to induce telomere uncapping, and 24 h later, with the indicated ASO or PSB. (**a**) liver and (**c**) kidney sections were stained for the indicated DDR markers. Scale bars, 10 μm. (**b**,**d**) Quantification of the data presented in **a** and **c**. Dot plots show the intensity of DDR foci per cell in (**b**) liver and (**d**) kidney (a.u.=arbitrary units). Lines depict the mean±s.e.m. *n*=4 mice per group; at least 200 cells per sample have been analysed; ***P<0.001, one-way analysis of variance with Sidak corrections for multiple comparisons.

ASOs have been successfully used to treat a number of different diseases; many of them are being tested in clinical trials and some have already been approved for use in the clinics^35^,^36^. To validate the efficacy of anti-teloG and anti-teloC ASOs in a living organism, we used an inducible *Trf2* knockout mouse model in which, following tamoxifen administration, TRF2 expression is lost, leading to telomere deprotection and a very robust telomeric DDR activation (Fig. 5a,b). After tamoxifen administration, but before detectable DDR induction, mice received a systemic dose of anti-teloG or anti-teloC ASOs, or phosphate-buffered saline (PBS) as control, by intraperitoneal (i.p.) injection (Supplementary Fig. 6c). Four days later, when telomeric DDR was expected to reach its maximal activation, organs were collected and tissue sections studied for DDR activation by immunofluorescence. We observed a marked reduction in foci intensity of both 53BP1 and the ATM target pKAP1 (ref. 37) in mice treated with both ASOs against tDDRNAs, compared with the control-treated mice, while γH2AX foci were unaffected. This striking effect could be observed in both organs studied: liver (Fig. 5a,b) and kidney (Fig. 5c,d). In summary, these unprecedented results demonstrate that sequence-specific targeting of tDDRNAs by ASOs allows telomeric DDR inhibition in both cultured cells and living mammals.

## Discussion

Our results show that dysfunctional telomeres are actively transcribed to generate double-stranded telomeric dilncRNAs and DDRNAs. While G-rich transcripts (TERRA) have previously been widely studied, this is the first report on the generation, processing and function of C-rich telomeric transcripts in mammals to our knowledge. Very low-abundant signals were detected for C-rich telomeric RNAs also in ref. 18. The observation that *Schizosaccharomyces pombe* strains deleted for telomeric proteins Taz1 or Rap1 (yeast homologues of the mammalian shelterin components TRF1/2 and RAP1, respectively) also show an induction of C-rich transcripts^38^,^39^ suggests that the observed induction is evolutionarily conserved. These results are consistent with RNA polymerase II recruitment at DSBs sites in fission yeast^31^, and with the reported increased RNA polymerase II occupancy at telomeres on TRF2 downregulation in human cells^40^.

The mechanism by which the proteins involved in the DDR are recruited to dysfunctional telomeres in a tDDRNA-dependent manner remains to be further investigated, although the ability of 53BP1 to bind RNA through its Tudor domain^41^ can be involved.

The evidence that only DDRNAs bearing a telomeric sequence are able to restore DDR activation at telomeres following RNase A treatment, and that only ASOs against tDDRNAs inhibit DDR at telomeres, but not at DSBs randomly generated at various genomic locations by IR, strongly suggests that tDDRNAs act in a locus- and sequence-specific manner.

Finally, we propose that the ability to detect tDDRNAs and to inhibit telomeric transcripts *in vitro* and *in vivo* by ASOs may have important implications in human physiology and pathology. tDDRNAs may be important biomarkers of DDR activation on telomeric damage, an event associated with physiological ageing and cancer^11^,^12^,^13^,^14^,^15^,^16^. In addition, many medical conditions, collectively known as telomere syndromes, and some progeric syndromes, are caused by accelerated telomere shortening and dysfunction with consequent DDR activation^17^. It is therefore conceivable that tDDRNA detection and modulation, for instance by ASO inhibition, can be exploited to address pathological conditions caused by telomere dysfunction.

## Methods

### Cell culture

Rosa26-CreERT2 *Trf2*^*F/F*^ and *Trf2*^*F/+*^ (ref. 20) MEFs, a gift from Eros Lazzerini Denchi (The Scripps Research Institute, La Jolla, USA), were grown in DMEM supplemented with 10% fetal bovine serum and 1% glutamine; for CreER activation, cells were treated with of 4OHT (600 nM) and analysed 48–72 h later. MEFs *Rosa26-CreERT2 Trf2*^*F/F*^ eGFP-TRF1 were obtained by retroviral infection of *Rosa26-CreERT2 Trf2*^*F/F*^ using eGFP-TRF1 pWzl-Hygro plasmid, a gift from Titia de Lange (Addgene plasmid # 19834), and selected with Hygromycin (200 μg ml^−1^). T19 fibrosarcoma cells^24^, a gift from Titia de Lange (The Rockefeller University, New York, USA), were grown in DMEM supplemented with 10% fetal bovine serum, 1% glutamine and doxycycline (100 ng ml^−1^). For induction, cells were cultured without doxycycline for 7–8 days. All cell lines used in this study were negative for mycoplasma contaminations.

### Animals and treatments

Rosa26-CreERT mice (Jackson Laboratories) and *Trf2* conditional knockout mice^33^ and mice carrying a p53 conditional allele (Jackson Laboratories) were crossed to generate Trf2/p53/Rosa26 mice. Mice were maintained in 129/c57Bl6 genetic background. All mice were bred and maintained under pathogen-free condition at the Scripps Research Institute, La Jolla, USA, and were handled according to Institutional Animal Care and Use Committee guidelines. To activate CreER, 8–10-week-old mice (both males and females) were injected i.p. with tamoxifen dissolved in sunflower oil or with vehicle at a final concentration of 75 mg kg^−1^. After 24 h, ASOs dissolved in PBS were administrated by i.p. at concentration of 15 mg kg^−1^. Mice were sacrificed after 5 days post tamoxifen injection. Tissues were collected and frozen in dry ice and embedded in optimal cutting temperature (OCT) tissue TEC (Sakura).

No power analysis was done to choose the size of the samples. No specific method of randomization was used to assign groups. Animals were assigned to experimental groups so as to minimize the influence of other variables such as age or sex on the outcome. No blinding approach was used for the experiments.

### RNA isolation

Total RNA from cultured cells was extracted with RNeasy Mini Kit (Qiagen) for messenger RNA detection, or with mirVana miRNA Isolation kit (Life Technologies) for DDRNA and dilncRNA detection, according to the manufacturer’s instructions. RNA extracted with mirVana miRNA Isolation kit using the Enrichment Procedure for Small RNAs was used as starting material for Target Enrichment experiments.

### Real-time quantitative PCR

A measure of 1 μg of total cell RNA was reverse transcribed using SuperScript VILO cDNA Synthesis Kit. A volume corresponding to 10 ng of initial RNA was used for each qPCR reaction using GoTaq qPCR Master Mix (Promega) on a Roche LightCycler 480 sequence detection system. Each reaction was performed in triplicate. Human ribosomal protein large P0 (Rplp0) and mouse beta-2 microglobulin (B2M) were used as control transcripts for normalization. Primers sequences (5′–3′ orientation) were:

mDicer Fw GCAAGGAATGGACTCTGAGC

mDicer Rv GGGGACTTCGATATCCTCTTC

mDrosha Fw CGTCTCTAGAAAGGTCCTACAAGAA

mDrosha Rv GGCTCAGGAGCAACTGGTAA

mB2M Fw CTGCAGAGTTAAGCATGCCAGTA

mB2M Rv TCACATGTCTCGATCCCAGTAGA

hDicer Fw GCAAAGCAGGGCTTTTCAT

hDicer Rv AGCAACACAGAGATCTCAAACATT

hDrosha Fw TGCACACGTCTAACTCTTCCAC

hDrosha Rv GGCCCGAGAGCCTTTTATAG

hRplp0 Fw TTCATTGTGGGAGCAGAC

hRplp0 Rv CAGCAGTTTCTCCAGAGC

### Real-time quantitative PCR for small RNAs

A measure of 5 μg of total cellular RNA were fractionated on a 10% polyacrylamide, 7 M Urea gel and RNA species shorter than 40 nucleotides were gel-extracted. A measure of 10 pg of an RNA spike-in was added to all samples before cellular RNA was loaded on the gel, to normalize for the efficiency of RNA extraction from gel. complementary DNA (cDNA) was synthesized using the miScript II RT kit (Qiagen) with HiSpec buffer. PCR was performed using the miScript PCR system (Qiagen) accordingly to the manufacturer’s instructions. Each reaction was performed in triplicate. mir29b was used as a control gene for normalization. Primer sequences (5′–3′ orientation):

mir29b TAGCACCATTTGAAATCAGTGTT

spike-In CGAATTCCACAAATTGTTATCC

teloG TAGGGTTAGGGTTAGGGT

teloC CCCTAACCCTAACCCTAA

### Strand-specific real-time quantitative PCR

Samples were treated with DNase I (Thermo Scientific) at 37 °C for 1 h to remove any potential residual genomic DNA contamination (one unit of DNase I per 1 μg of RNA). A measure of 1 μg of total RNA was reverse-transcribed using the Superscript First Strand cDNA synthesis kit (Invitrogen) with strand-specific primers. To amplify telomeric repeats, we adapted a technique described in ref. 25, which allows the generation of a fixed-length amplification product. For reverse transcription, we used the following primers: RPP0 Rv for the detection of Rplp0 messenger RNA; teloC Rv for the detection of G-rich telomeric precursor; and teloG Rv for the detection of C-rich telomeric precursor. qPCR was performed using SYBR green (Roche). For each reaction, 50 ng of cDNA were used. Each reaction was performed in triplicate. Rplp0 was used as a control gene for normalization. Primer sequences (5′–3′ orientation) were:

RPP0 Fw TTCATTGTGGGAGCAGAC

RPP0 Rv CAGCAGTTTCTCCAGAGC

teloC Rv CCCTAACCCTAACCCTAA

teloG Rv TAGGGTTAGGGTTAGGG

telo Fw CGGTTTGTTTGGGTTTGGGTTTGGGTTTGGGTTTGGGTT

telo Rv GGCTTGCCTTACCCTTACCCTTACCC TTACCCTTACCCT

### Targeted sequencing of small RNA

Two linkers were ligated to the two ends of the RNA molecules in the sample to be analysed. The 3′-end of the starting RNA was ligated to a monoadenylated DNA linker by a T4 RNA ligase 2-truncated enzyme (NEB) incubated for 1 h at 25 °C. The 5′ RNA linker was then ligated by a T4 RNA ligase 1 (NEB) to the target RNA at 20 °C for 1 h, after removing the 5′ cap structure by Tobacco Acid Pyrophosphatase (Epicentre), incubated at 37 °C for 1 h. Linkers enabled cDNA synthesis using PrimeScript RT–PCR Kit (Takara). The reverse transcription reaction was incubated at 44 °C for 1 h. Subsequent PCR amplification using Phusion High-Fidelity DNA Polymerase (NEB) was carried out as follows: 98 °C 2 min; 22 cycles of: 98 °C for 30 s, 55 °C for 30 s, 72 °C for 30 s; 72 °C for 5 min; hold at 4 °C. To capture the amplified cDNA targets, complementary RNA baits containing biotin-labelled nucleotides were used. These RNA baits were produced by using AMbion MAXIscript T7 *In Vitro* Transcription kit (Life Technologies) and Biotin RNA labelling Mix (Roche). A T7-promoter-containing double-stranded DNA was incubated at 37 °C for 1 h to allow for *in vitro* transcription. RNA baits and cDNA targets were incubated at 37 °C for 48 h in the presence of SUPERase-inhibitor (Life Technologies) and of the following blocking agents: Human Cot-1 (Life Technologies), UltraPure Salmon Sperm DNA Solution (Thermo Scientific) and a 200 μM Customized Block. The hybrid RNA–cDNA molecules were captured by Dynabeads MyOne Streptavidin C1 (Life Technologies) beads, while non-targeted cDNAs were washed away. Captured cDNAs were then barcoded by PCR with Script Index PCR primers (Illumina) and sequenced by a MiSeq (Illumina) sequencer. Oligonucleotide sequences (5′–3′ orientation) were:

3′-DNA linker AGATCGGAAGAGCACACGTCTGAACTCCAGTCAC-Amine

5′-RNA linker ACACUCUUUCCCUACACGACG CUCUUCCGAUCU

RT primer GTGACTGGAGTTCAGACGTGTG CTCTTCCGATCT

PCR Fw AATGATACGGCGACCACCGAGA TCTACACTCTTTCCCTACACGACGCTCTTCCGATCT

PCR Rv CAAGCAGAAGACGGCATACGAGATCGGTCTCGGCATTCCTGCTGAACCGCTCTTCCGATCT

Block Fw AATGATACGGCGACCACCGAGATCTACACTCTTTCCCTACACGACGCTCTTCCGATCT

Block Rv CAAGCAGAAGACGGCATACGAGATCGTGATGTGACTGGAGTTCAGACGTGTGCTCTTCCGATCT

### Analysis of small RNA sequencing data

Illumina adapters and linkers used in the targeted sequencing of small RNA were trimmed using Trim Galore! (http://www.bioinformatics.babraham.ac.uk/projects/trim_galore/), discarding reads that became shorter than 10 nucleotides of length after trimming. Reads were mapped to a telomeric contig made of 15 consecutive TTAGGG repeats and in parallel to the mouse genome (GRCm38/mm10) using Bowtie2 (ref. 42) with a very sensitive local option (-D 20 -R 3 -N 0 -L 20 -i S,1,0.50). Strand information was preserved to divide the aligned reads according to the original strand. Reads were parsed using SAMtools^43^ and *ad hoc* bash and perl scripts. To avoid spurious reads contamination, telomeric reads with mismatches or soft clipped portions were removed with *ad hoc* R scripts.

### RNA smFISH

Cells were fixed in 4% paraformaldehyde (PFA) for 10 min at room temperature (RT) and permeablized overnight at 4 °C using 70% ethanol. Cells were rehydrated in a solution containing 20% formamide and 2 × SSC for 5 min and then treated with 10 nM FISH probes for 12 h in 2 × SSC containing 10% dextran sulfate, 2 mM vanadyl–ribonucleoside complex, 0.02% RNase-free BSA, 1 μg μl^−1^ *E. coli* tRNA and 20% formamide at 37 °C. After hybridization, the cells were washed twice for 30 min at 37 °C using a wash buffer (20% formamide in 2 × SSC). Cells were then mounted in solution containing 10 mM Tris/HCl, 2 × SSC, 2 mM trolox, 5 mM protocatechiuc acid and 50 nM protocatechuate dehydrogenase. HILO imaging was done, as described^26^. FISH signals were detected using custom-written macros in ImageJ that were based on the analysis routine as described^44^. To calculate the relative intensity of FISH spots, a 21 × 21 pixel area around the FISH signal locus was normalized with the intensity of a 21 × 21 pixel area outside of the locus. Intensity of 21 × 21 pixel area outside the cell was used for background subtraction. Red and green channels were corrected for signal bleed-through–samples that were labelled only with Cy5 or GFP were imaged using all excitation lines (488 and 640 nm) and signal arising from non-cognate excitation were used as a correction factor. All probes were tested for RNA specificity by visualizing the loss of FISH signal on RNase A treatment. For these control experiments cells were treated with RNase A (1 μg μl^−1^) in DPBS at 37 °C, before probe treatment. FISH probes were purchased as high-performance liquid chromatography purified DNA oligonucleotides from IDT. Probe sequences were as follows (5′–3′ orientation):

TeloG: CCTAACCCTAACCCTAACCCTAAC-Cy5

TeloC: GGTTAGGGTTAGGGTTAGGGTTAG-Cy5

### Combined IF/RNA smFISH

MEFs *Rosa26-CreERT2 Trf2*^*F/F*^ eGFP-TRF1 were fixed in 4% PFA for 10 min and treated with a solution containing 1:1 methanol:acetic acid to irreversibly photobleach eGFP-TRF1. Cells were then washed three times in PBS for 5 min each and the standard immunofluorescence protocol was continued. In addition, all blocking, primary and secondary antibody incubations were performed in 1 × PBBR (1% Ultrapure/RNase-free BSA+0.1 U μl^−1^ rRNAsin, 1 mM EDTA in PBS). Importantly, the Human-anti-centromere antibody was purified by passing through G25 spin column (Millipore), which was pre-equilibrated in 1 × PBS with 5% glycerol and 0.1 U μl^−1^ rRNAsin. Cells were fixed again in in 4% PFA (in 1 × PBS) for 10 min after immunofluorescence protocol to stably retain the bound primary and secondary antibodies and the standard RNA smFISH protocol was continued.

### Ionizing radiation

IR (1 Gy) was generated by a high-voltage X-ray-generator tube (Faxitron X-Ray Corporation).

### Transfection

Transfections were carried out with Lipofectamine RNAiMAX (Invitrogen) according to the manufacturer’s instructions.

### siRNA

ON-TARGETplus SMARTpool short interfering RNA (siRNA) oligonucleotides (Dharmacon) were used at a final concentration of 5–20 nM. Sequences were as follows:

siControl (siGFP) GCAAGCUGACCCUGAAGUUCAU

siDicer human UAAAGUAGCUGGAAUGAUG;

GGAAGAGGCUGACUAUGAA; GAAUAUCGAUCCUAUGUUC;

GAUCCUAUGUUCAAUCUAA

siDrosha human CAACAUAGACUACACGAUU;

CCAACUCCCUCGAGGAUUA; GGCCAACUGUUAUAGAAUA;

GAGUAGGCUUCGUGACUUA

siDicer1 mouse GGUAGACUGUGGACCGUUU;

GGAAAUACCUGUACAACCA; GCAAUUUGGUGGUUCGUUU;

ACAGGAAUCAGGAUAAUUA

siDrosha mouse UGGAAGGAGUUACGCUUUA;

GGAAUCCGCCACAGCAUUU; GUGAUCACUUUCCCGAUUA;

UAAUGCACCUGGACAAGUU

### ASOs sequences

The locked nucleic acid mixmer oligonucleotides with a fully phosphorothioate backbone were produced by Exiqon. They were used at a final concentration of 20 nM for transfection of cultured cells, 15 mg kg^−1^ for mouse injections. Sequences were as follows (5′–3′ orientation):

Control ACTGATAGGGAGTGGTAAACT

anti-teloG CCTAACCCTAACCCTAACCC

anti-teloC GGGTTAGGGTTAGGGTTAGGG

### Immunoblot

Cells were lysed in Laemmli sample buffer (2% SDS, 10% glycerol, 60 mM Tris-HCl pH 6.8). A measure of 50 μg of whole cell extracts were resolved by SDS–polyacrylamide gel electrophoresis. Proteins were transferred to nitrocellulose membrane, which was blocked in 5% milk in TBST (0.1% Tween in tris-buffered saline), and incubated with the primary antibody for 1 h at RT, and with a horseradish peroxidase-conjugated secondary antibody for 1 h at RT. Quantification of protein bands was done by ImageJ software, subtracting the background signal and normalizing for the housekeeper. Uncropped scans of the blots are provided in Supplementary Fig. 7.

### Immunofluorescence for cultured cells

Cells were fixed with 4% PFA or 1:1 methanol/acetone solution. After incubation with blocking solution, cells were stained with primary antibody for 1 h at RT, washed and incubated with secondary antibodies for 40 min at RT. Nuclei were stained with 4,6-diamidino-2-phenylindole (DAPI; 1 μg ml^−1^). Samples were mounted in mowiol or in imaging solution as in RNA smFISH.

### Immunofluorescence for mouse tissues

A measure of 4-μm tissue sections were fixed for 10 min in 4% PFA, incubated in blocking solution (2% BSA, 0.1% Tween in PBS) for 1 hour at RT. Then sections were incubated for 1 h at RT with primary antibodies, washed in blocking solution and incubated for 1 h at RT with secondary antibody. Nuclei were stained with DAPI (1 μg ml^−1^). Samples were mounted with glycerol solution.

### Metaphase spreads

MEFs were incubated for 2 h with 0.2 μg ml^−1^ colcemid (Gibco). The cells were collected by trypsinization, resuspended in 0.075 M KCl at 37 °C for 30 min, and fixed overnight in methanol/acetic acid (3:1) at 4 °C. The cells were dropped onto glass slides and the slides were dried overnight. The next day, the slides were rehydrated with PBS for 5 min. Slides were incubated consecutively with 75, 95, and 100% ethanol and allowed to air dry before FISH.

### DNA FISH

Cultured cells after immunofluorescence were fixed with 4% PFA, 0.1% Triton X-100 for 10 min at RT, then incubated with 10 mM glycine for 30 min at RT. Interphase or metaphase cells were incubated with a telomeric PNA probe (0.5 μM, TelC-Cy3 from PANAGENE, catalogue number: F1002-5) in a buffer containing 70% formamide, 1 mg ml^−1^ blocking reagent (Roche), 10 mM Tris-HCl pH 7.4 and the coverslips were denatured on a heat block 5 min at 80 °C and incubated for 2 h in the dark. The coverslips were washed twice with 70% formamide, 10 mM Tris-HCl pH 7.4 for 15 min each and three times with 100 mM Tris-HCl pH 7.4, 0.15 M NaCl, 0.08% Tween 20 for 5 min each. DNA was stained with DAPI (1 μg ml^−1^). Samples were mounted in mowiol.

### *In situ* PLA

Cells were labelled according to the manufacturer's instructions (Sigma). Briefly, MEFs *Rosa26-CreERT2 Trf2*^*F/F*^ eGFP-TRF1 were treated with 4OHT (600 nM). Forty-eight hours later cells were permeabilized with 0.6% Tween 20 in PBS for 20 min at RT and incubated with 7 ng μl^−1^ of Alexa Fluor 647-conjugated synthetic DDRNAs, in the presence of the RNase inhibitor RNaseOUT (1 U μl^−1^, Invitrogen) for 25 min at RT. Cells were then fixed with 4% PFA. After incubation with primary antibodies, appropriate PLA probes (secondary antibodies conjugated with oligonucleotides) were added to the samples. Ligation of the oligonucleotide probes that were in proximity (<40 nm) was then performed, after which fluorescently labelled oligonucleotides were added, together with a DNA polymerase to generate a signal detectable by a fluorescence microscope.

### Imaging

Images were acquired using a widefield Olympus Biosystems BX71 microscope or a Leica TCS SP2 AOBS confocal laser microscope. Number and intensity of foci per cell and number of dots per cell were analysed by the imaging software CellProfiler^29^, using the same pipeline for each sample in the same experiment. Percentages of DDR-positive cells were scored manually. A cell was counted as positive if showing >3 foci.

### Antibodies

Anti-γH2AX (Millipore, 05-636, 1:400; Cell Signalling, 9718, 1:1,000); anti-ATM pS1981 (Millipore, 05-740, 1:100); anti-pS/TQ (Cell Signalling, 2851, 1:200); anti-53BP1 (Novus, NB100-304, 1:200; Bethyl, A300-272A, 1:1,000; Santa Cruz, sc-22760, 1:1,000); anti-pKap1 (Bhetyl, A300-767A, 1:1,000); anti-Flag (Sigma, F3165, 1:500); anti-ATM (Sigma-Aldrich, A1106, 1:1,000); anti-H2AX (Abcam, ab11175, 1:1,000); anti-vinculin (Sigma-Aldrich, V9131, 1:10,000); anti-GFP (Abcam, ab290, 1:4,000); anti-Cy5 (Abcam, ab52061,1:500); anti-Centromere Protein (Antibodies Incorporated, 15-234-0001, 1:100).

### RNase A treatment on permeabilized living cells

Cells were permeabilized with 0.6% Tween 20 in PBS for 20 min at RT, and RNase A treatment was carried out in 1 mg ml^−1^ of ribonuclease A from bovine pancreas (Sigma-Aldrich R5503), or acetylated albumin from bovine serum (Sigma-Aldrich B8894), in PBS for 30 min at RT. For complementation experiments samples were washed with PBS, treated with RNase inhibitor RNaseOUT (1 U μl^−1^, Invitrogen) and α-amanitin (20 ng μl^−1^, Sigma-Aldrich A2263) for 15 min. Cells were incubated with total cell RNA or yeast tRNA (3 ng μl^−1^), rDICER products (1.5 ng μl^−1^) or synthetic dsRNA oligos (1–100 nM using yeast tRNA up to 100 nM) for 25 min at RT. Cells were then fixed with 4% PFA.

### Turbo DICER RNAs generation

DICER RNA products were generated as follows. pTH5 (ref. 45), a pSP73-backboned plasmid containing 27 telomeric repeats flanked by T7 and SP6 promoter, was used for *in vitro* transcription. SP6 RNA Polymerase (Promega) and T7 RNA Polymerase (Promega) were used together for *in vitro* transcription allowing the generation of a telomeric dsRNA. The DNA template was later removed by using RQ1 RNase-Free DNase (Promega) for 15 min at 37 °C. Acid-phenol:chloroform, pH 4.5 (Ambion) and subsequent ethanol precipitation was used to isolate the *in vitro*-transcribed RNA. The dsRNA was then incubated for 8 h at 37 °C using a Recombinant Human Turbo Dicer Enzyme Kit (Genlantis). To remove salts and undigested templates, RNA Purification Column 1 and 2 (Genlantis) were used. RNA products were quantified and checked on a 3% agarose gel. As a control, the same procedure was followed with a 1 kb construct containing the neomycin DNA sequence. Equal amounts of DICER RNA products generated in this way were used in complementation experiments with MEFs following RNase A treatment.

### Synthetic DDRNAs

3′-labelled Alexa Fluor 647 RNA oligonucleotides were resuspended in 1 × siRNA Buffer (Dharmacon). Equal amount of two oligonucleotides were mixed to generate double-stranded DDRNAs and treated as follows: 90 °C for 1 min, 70 °C for 2 min, 55 °C for 2 min, 37 °C for 5 min, RT for 5 min. Sequences were as follows (5′–3′ orientation):

Control pair UGUUAGCUGGAGUGAAAACUU

GUUUUCACAAAGCUAACACA

Telomeric pair AGGGUUAGGGUUAGGGUUAGG

UAACCCUAACCCUAACCCUAA

### Statistical analysis

Results are shown as mean±s.e.m. or s.d. or percentages±95% confidence interval as indicated. *P* value was calculated by the indicated statistical tests, using Prism software. Statistical significance of enrichment of small RNA species on telomere deprotection was calculated using the Fisher’s exact test. The differences in the fraction of 20–23 nucleotides versus total small RNAs were calculated by performing a one-tailed binomial test, where the null hypothesis is the fraction of 20–23 nucleotide reads in the control sample and the alternative hypothesis is the count of 20–23 nucleotide reads versus total small RNAs in the sample with telomere deprotection. The deviation from a theoretically expected distribution in a UUAGGG or CCCUAA context was calculated by performing a one-tailed binomial test, where the null hypothesis is the expected fraction of U, A, G (or A, U, C) nucleotides in the wild type (that is, 2/6, 1/6 and 3/6, respectively), and the alternative hypothesis is the observed proportion of each nucleotide.

In figure legends, *n* indicates the number of independent experiments.

### Data availability

The authors declare that all data supporting the findings of this study are available within the article and its Supplementary files or from the authors on a reasonable request. The sequencing data refer to the targeted sequencing of small RNA (Fig. 1b; Supplementary Figs 1g and 2a). These data are accessible through GEO (Gene Expression Omnibus) Series accession number GSE86964.

## Additional information

**How to cite this article:** Rossiello, F. *et al*. DNA damage response inhibition at dysfunctional telomeres by modulation of telomeric DNA damage response RNAs. *Nat. Commun.* **8,** 13980 doi: 10.1038/ncomms13980 (2017).

**Publisher’s note:** Springer Nature remains neutral with regard to jurisdictional claims in published maps and institutional affiliations.

## Supplementary Material

Supplementary Information Supplementary Figures
