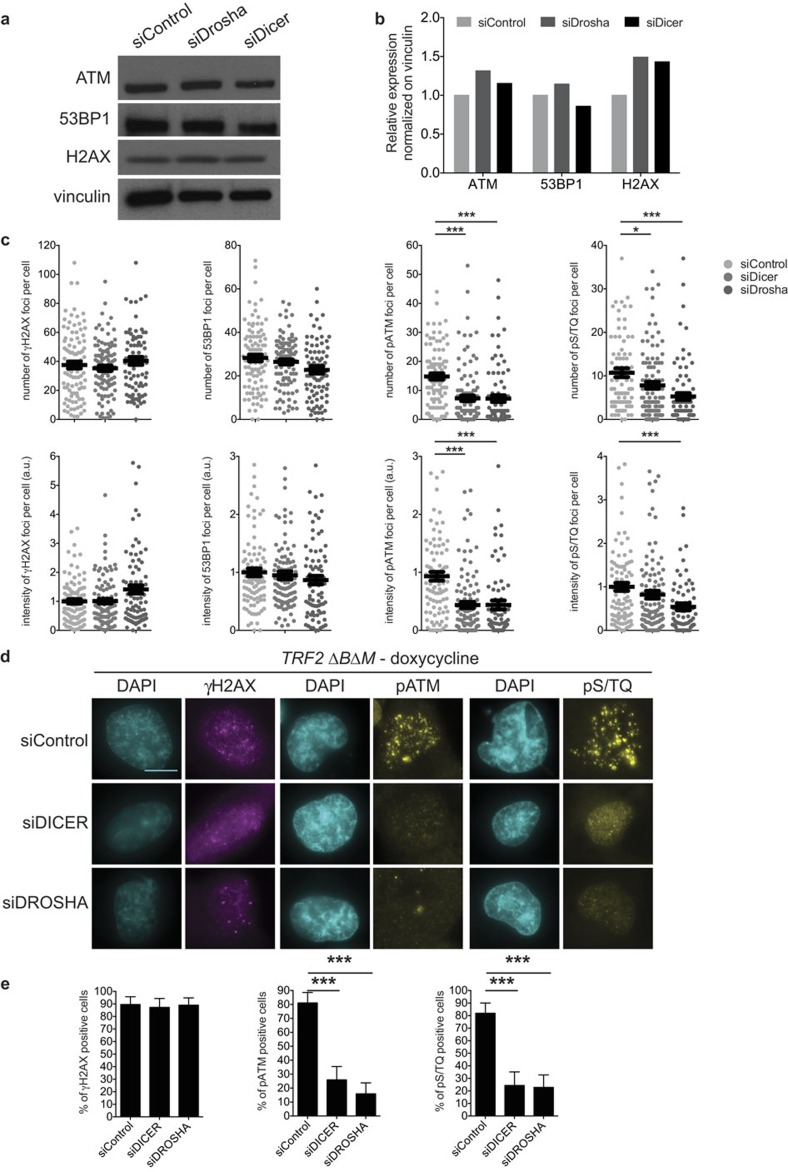# Corrigendum: DNA damage response inhibition at dysfunctional telomeres by modulation of telomeric DNA damage response RNAs

**DOI:** 10.1038/ncomms15344

**Published:** 2017-04-13

**Authors:** Francesca Rossiello, Julio Aguado, Sara Sepe, Fabio Iannelli, Quan Nguyen, Sethuramasundaram Pitchiaya, Piero Carninci, Fabrizio d'Adda di Fagagna

Nature Communications 8: Article number: 13980; DOI: 10.1038/ncomms13980 (2017); Published: 12 27 2017; Updated: 04 13 2017

In Supplementary Fig. 3d, the two columns of DAPI images associated with pATM and pS/TQ images were inadvertently swapped. The correct version of this figure appears below.

## Figures and Tables

**Figure 1:**