# Supplementary material for: DNA damage response inhibition at dysfunctional telomeres by modulation of telomeric DNA damage response RNAs
Source: Nat Commun. 2017 Feb 27;8:13980. doi: 10.1038/ncomms13980 (PMC5473644; doi:10.1038/ncomms13980)
Supplement: Supplementary Information — Supplementary Figures [file ncomms13980-s1.pdf]

Supplementary Figure 1

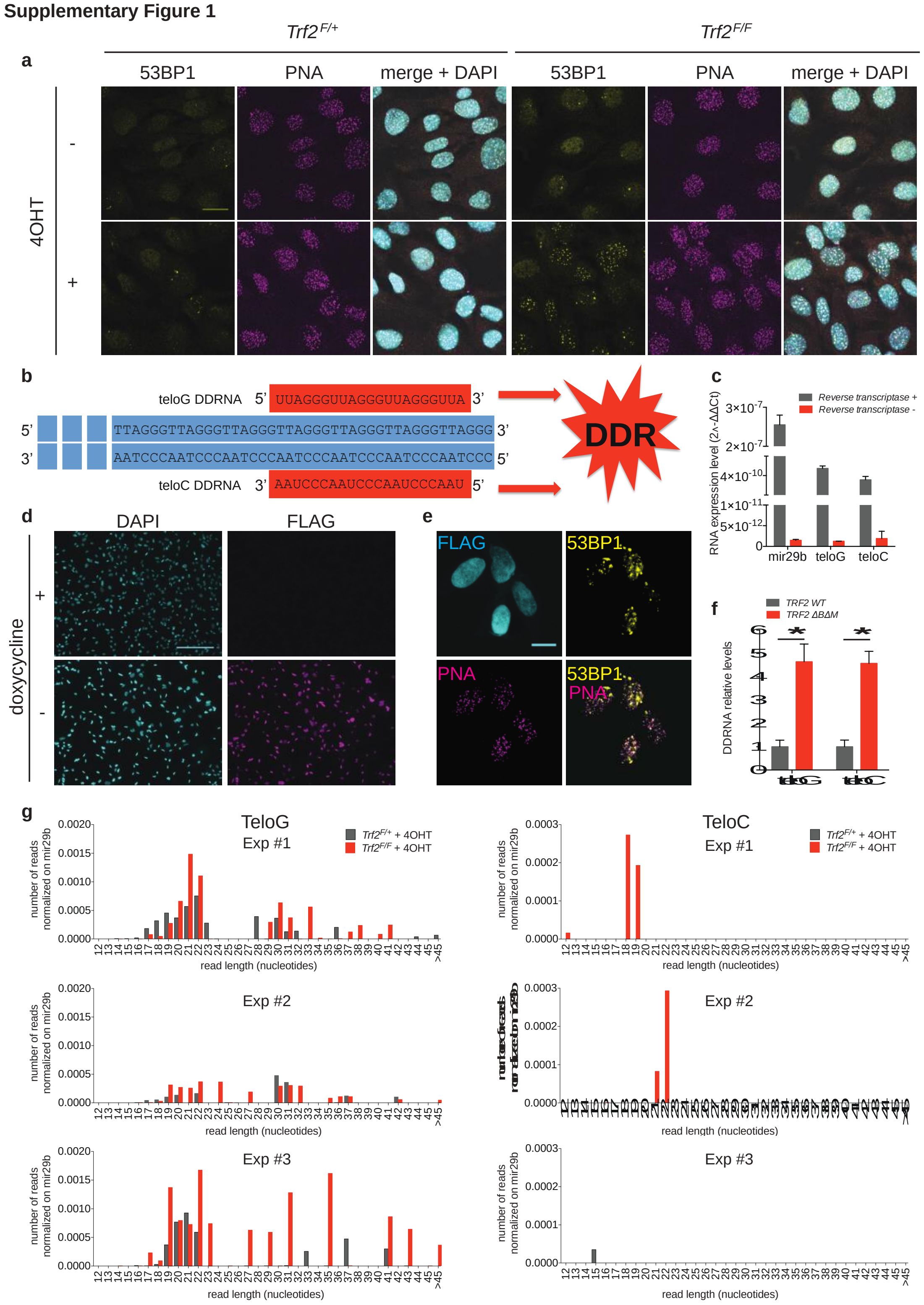

Supplementary Figure 1. (a) MEFs of the indicated genotype were treated, or not, with 4-hydroxytamoxifen (4OHT) for 48 hours and stained for the DDR marker 53BP1 and a telomeric PNA probe. Scale bar, 20 µm. **(b)** Schematic representation of a deprotected telomere and the tDDRNA species that are generated from the two DNA strands. **(c)** Gel-extracted small RNA fraction (< 40 nucleotides) was used for miScript PCR amplification. cDNA synthesis reactions were performed in the presence (Reverse transcriptase +) or absence (Reverse transcriptase -) of the reverse transcriptase. Error bars represent s.d. of 3 technical replicates **(d)** T19 cells were cultured in presence or absence of doxycycline. The expression of FLAG-tagged *TRF2 ΔBΔM* gene was evaluated by FLAG staining in cells cultured without doxycycline for 8 days. Scale bar, 200 µm. **(e)** T19 cells were cultured without doxycycline for 8 days and stained for FLAG, 53BP1 and a telomeric PNA probe to show DDR foci co-localizing with telomeres. Scale bar, 10 µm. **(f)** Total cell RNA was isolated from T19 cells cultured in the presence or absence of doxycycline for 8 days. Gel-extracted small RNA fraction (< 40 nucleotides) was used for miScript PCR amplification to specifically detect DDRNAs. Error bars represent the s.e.m. n = 3 independent experiments. ***** Pvalue < 0.05, Student's t-test. **(g)** Histograms show the number of telomeric reads, separated by length, for both G- and C-rich reads normalized on mir29b1 reads for each single experiment described in Fig. 1b.

Supplementary Figure 2

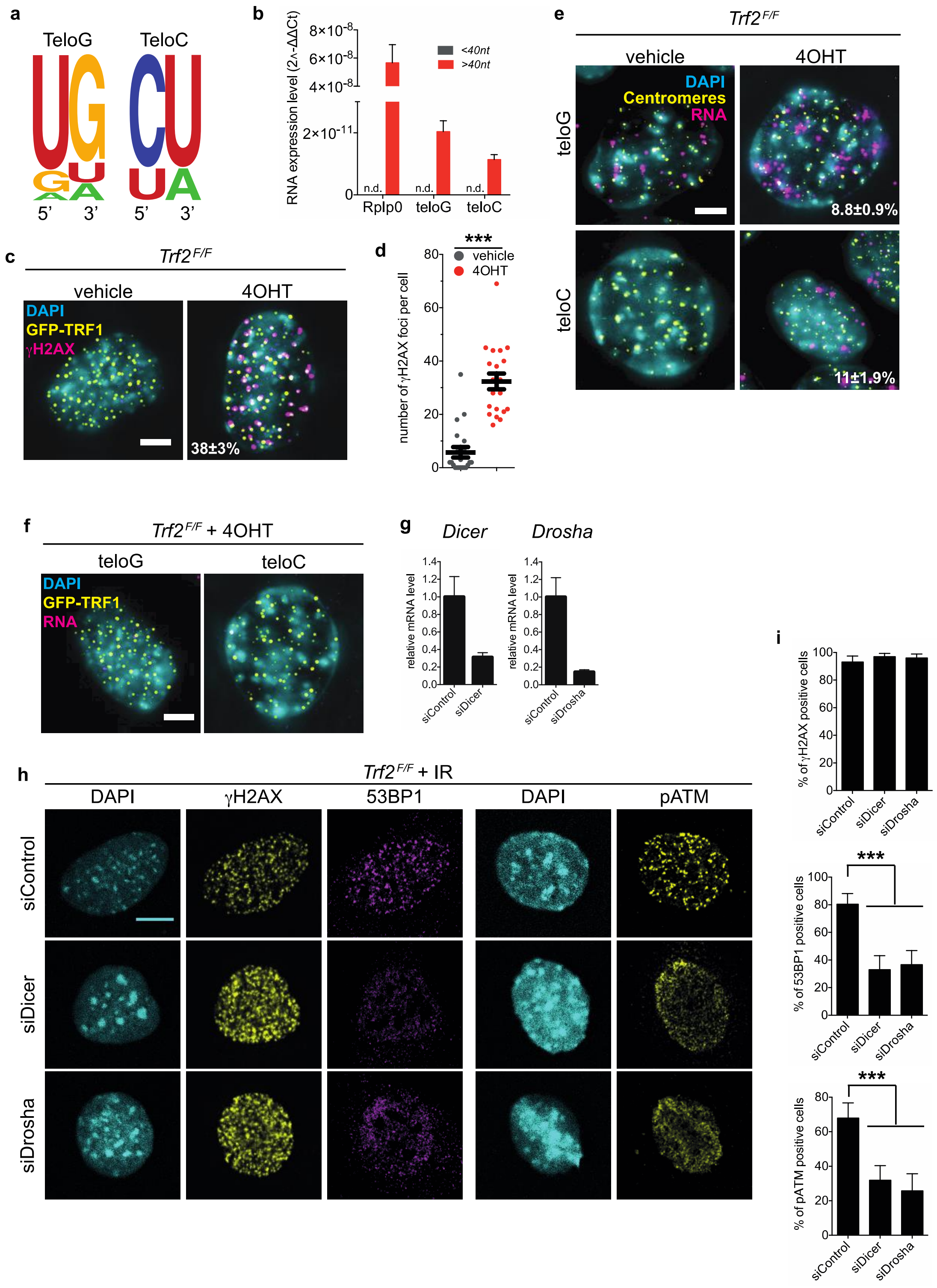

Supplementary Figure 2. (a) Logo plots show for each nucleotide the percentage of occurrences at the 5’ and the 3’ end of the 20-23 nucleotide-long reads. TeloG reads showed a preference for a U at their 5' and a G at their 3' end; TeloC reads showed a preference for a C/U at their 5' and a U at their 3' end. Pvalue < 0.001, binomial test. (**b**) Gel-extracted total RNA was fractionated in less than 40 (<40) and more than 40 (>40) nucleotides and used for strand specific RT-qPCR (n.d. = not detectable). Error bars represent s.d. of 3 technical replicates **(c-f)** MEFs *Trf2*$^{F/F}$ expressing GFP-TRF1 were treated with vehicle or 4-hydroxytamoxifen (4OHT) and analysed 48 hours later. **(c)** Representative images of γH2AX and GFP-TRF1 signals. The indicated numbers show the percentage of γH2AX signals co-localizing with GFP-TRF1 ± s.e.m. Scale bar, 5 µm **(d)** Quantification of data presented in panel **c**. Lines depict the mean number of foci per cell ± s.e.m. n = 2 independent experiments; at least 20 cells per sample were analysed; *** Pvalue < 0.001, Student’s t-test. **(e)** Representative images of cells probed for teloG and teloC telomeric transcripts (RNA) and stained for centromeres, using anti-centromere antibody. The indicated number shows the percentage of RNA signals co-localizing with centromeres ± s.e.m. Scale bar, 5 µm. **(f)** Representative images of RNase A-treated cells probed for teloG and teloC telomeric transcripts (RNA). Scale bar, 5 µm **(g)** Representative RT-qPCR to detect *Drosha* and *Dicer* mRNA levels upon siRNA treatment in MEFs *Trf2*$^{F/F}$. Error bars represent the s.e.m. of 3 technical replicates. **(h)** MEFs *Trf2*$^{F/F}$ were transfected with the indicated siRNA, were irradiated with 1 Gy 48 hours later and analysed after 10-60 minutes. Scale bar, 10 µm. **(i)** Quantification of data presented in panel **h**. Bar graphs show the percentage of DDR-positive cells ± 95% c.i. At least 100 cells per sample have been analysed; *** Pvalue < 0.001, chi-square test.

Supplementary Figure 3

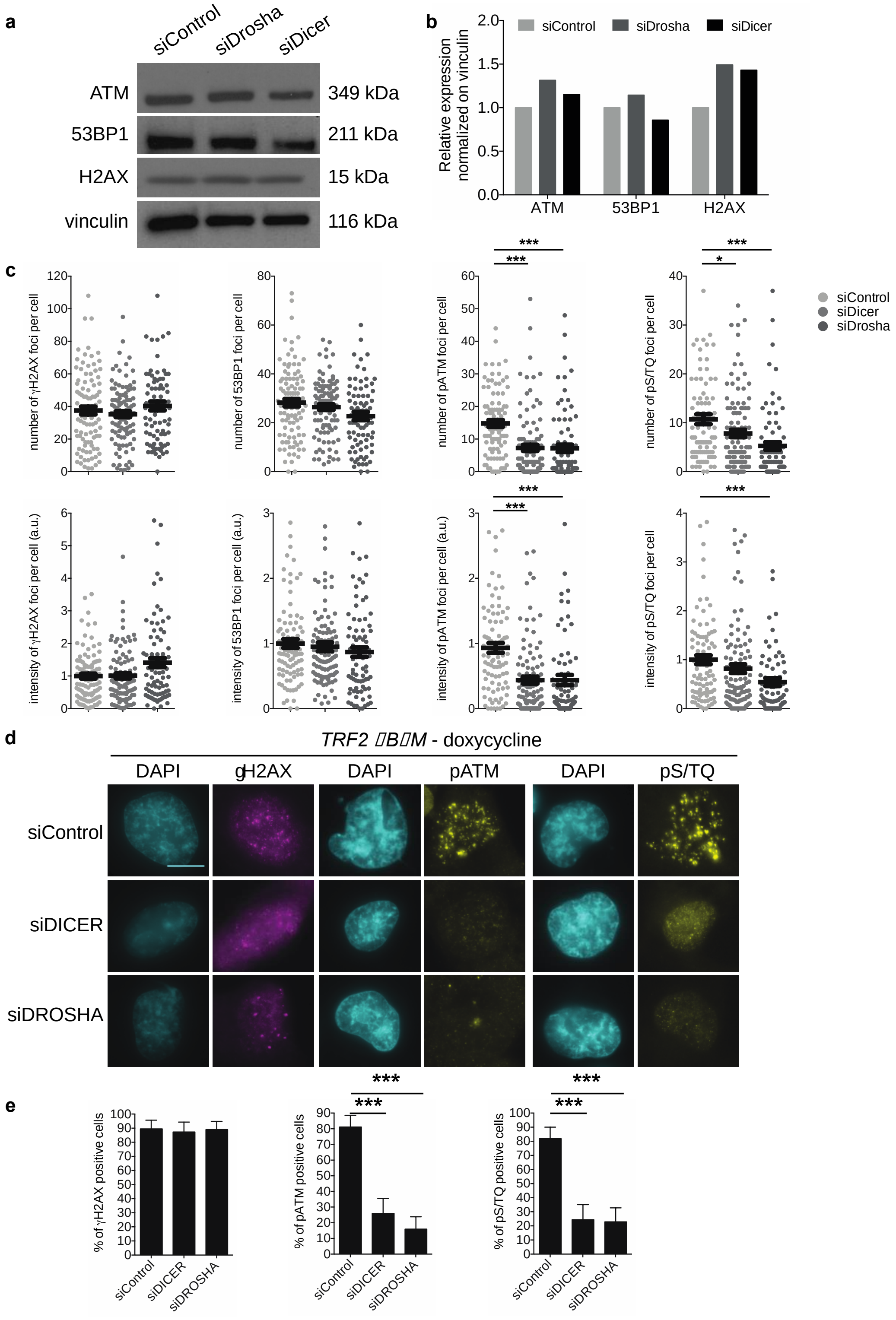

Supplementary Figure 3. (a) Representative immunoblot showing DDR protein levels in MEFs *Trf2*$^{F/F}$ upon *Drosha* or *Dicer* knockdown. **(b)** Quantification of bands shown in panel **a**. **(c)** Automated quantification by CellProfiler software of data presented in Figure 2c. Dot plots show the number or intensity of DDR foci per cell (a.u. = arbitrary units). Lines depict the mean ± s.e.m. n = 3 independent experiments; at least 100 cells per sample have been analysed; * Pvalue < 0.05, *** Pvalue < 0.001, Student's t-test. **(d)** T19 cells expressing TRF2 ΔBΔM were transfected with the indicated siRNA and stained for the indicated DDR markers. Scale bar, 5 μm. **(e)** Quantification of data presented in **d**. Bar graphs show the percentage of DDR-positive cells ± 95% c.i. n = 2 independent experiments; at least 100 cells per sample have been analysed; *** Pvalue < 0.001.

Supplementary Figure 4

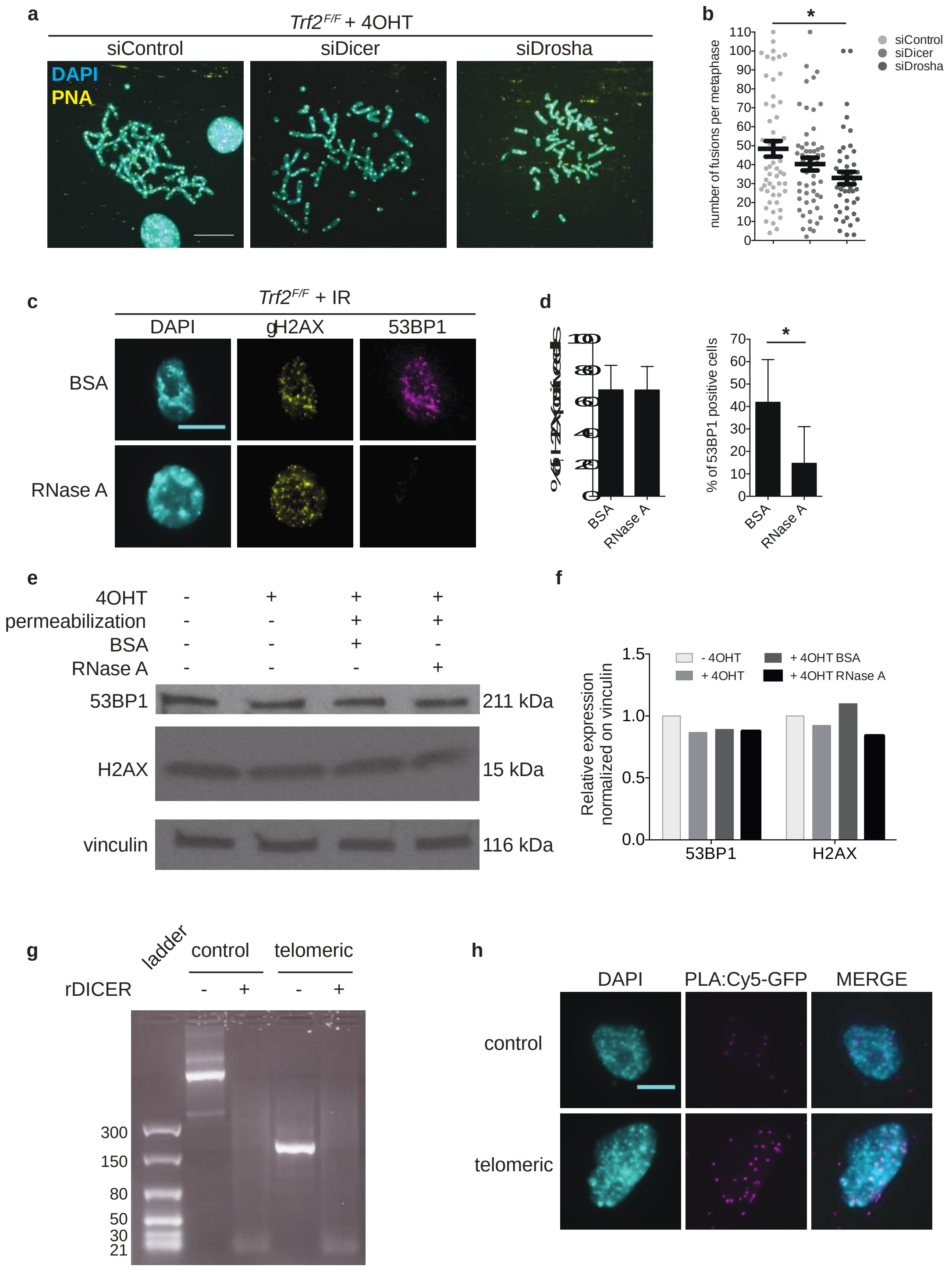

Supplementary Figure 4. (a) MEFs *Trf2*$^{F/F}$ were treated with 4-hydroxytamoxifen (4OHT) and transfected with the indicated siRNA. Metaphase spreads were stained with a telomeric PNA probe. Scale bar, 20 μm. **(b)** Quantification of data presented in panel **a**. Dot plots show the number of chromosomal fusions per metaphase. Lines depict the mean ± s.e.m. n = 5 independent experiments; at least 50 metaphases per sample have been analysed; * Pvalue < 0.05, Student's t-test. **(c)** MEFs *Trf2*$^{F/F}$ were treated with ionizing radiation (IR, 1 Gy), permeabilized 30 minutes later, treated with BSA or RNase A, and stained for the indicated DDR markers. Scale bar, 20 μm. **(d)** Quantification of data presented in panel **c**. Bar graphs show the percentage of DDR-positive cells ± 95% c.i. At least 40 cells per sample have been analysed; * Pvalue < 0.05, chi-square test. **(e)** Representative immunoblot showing DDR protein levels in MEFs *Trf2*$^{F/F}$ upon the indicated treatments. **(f)** Quantification of bands shown in panel **e**. **(g)** Agarose gel showing the length of double-stranded RNAs, generated upon recombinant DICER cleavage. **(h)** Representative images showing interaction between eGFP-TRF1 (detected by anti-GFP antibody) and the indicated DDRNA (detected by anti-Cy5 antibody), as measured by proximity ligation assay (PLA). Scale bar, 10 μm.

Supplementary Figure 5

a

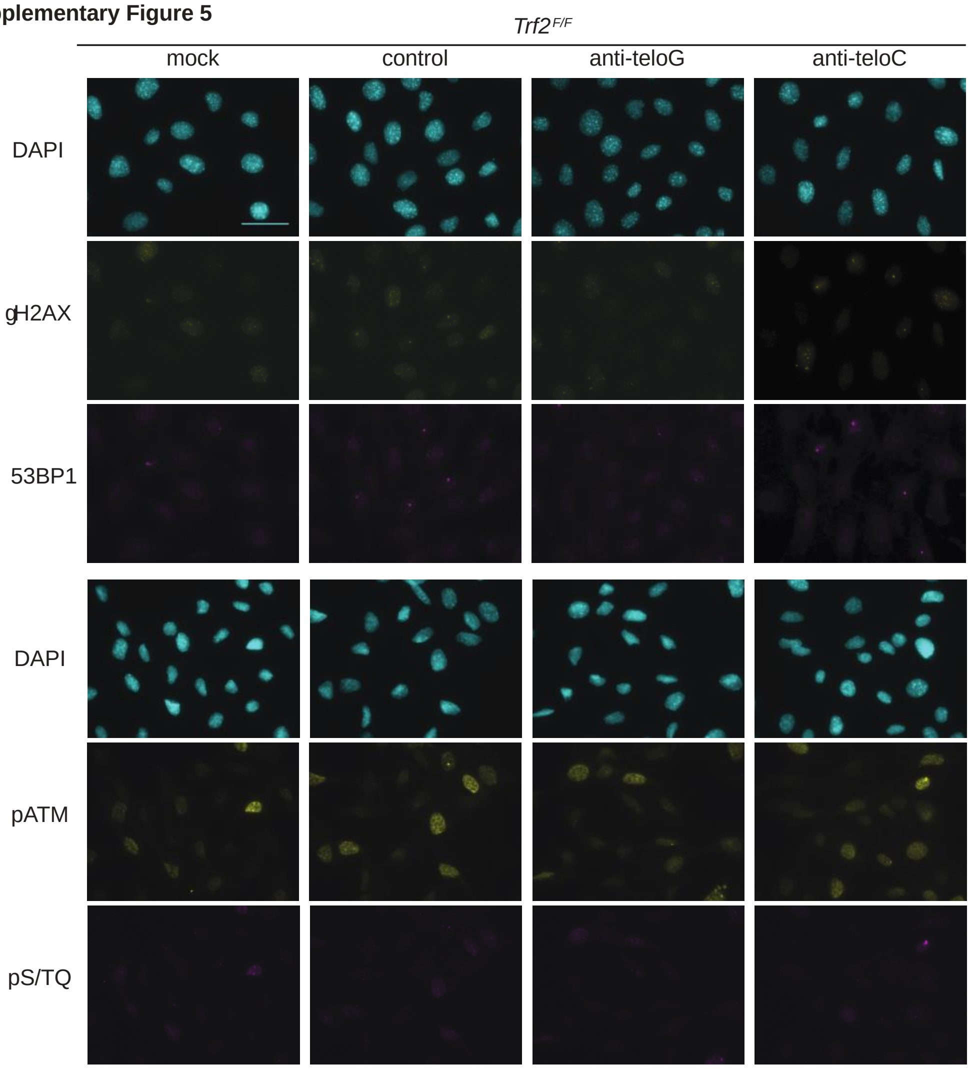

b

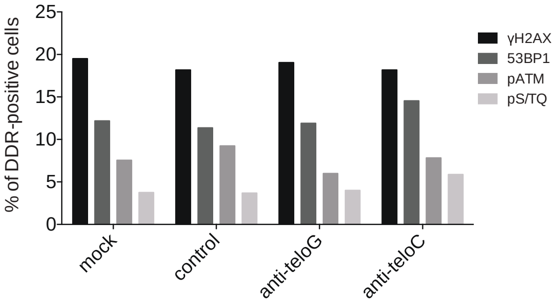

Supplementary Figure 5. (a) MEFs *Trf2*$^{F/F}$ were transfected with the indicated ASO and stained for the indicated DDR markers 48 hours later. Scale bar, 50 μm. **(b)** Quantification of data presented in panel **a**. Bar graph shows the percentage of DDR-positive cells. At least 40 cells per sample have been analysed.

Supplementary Figure 6

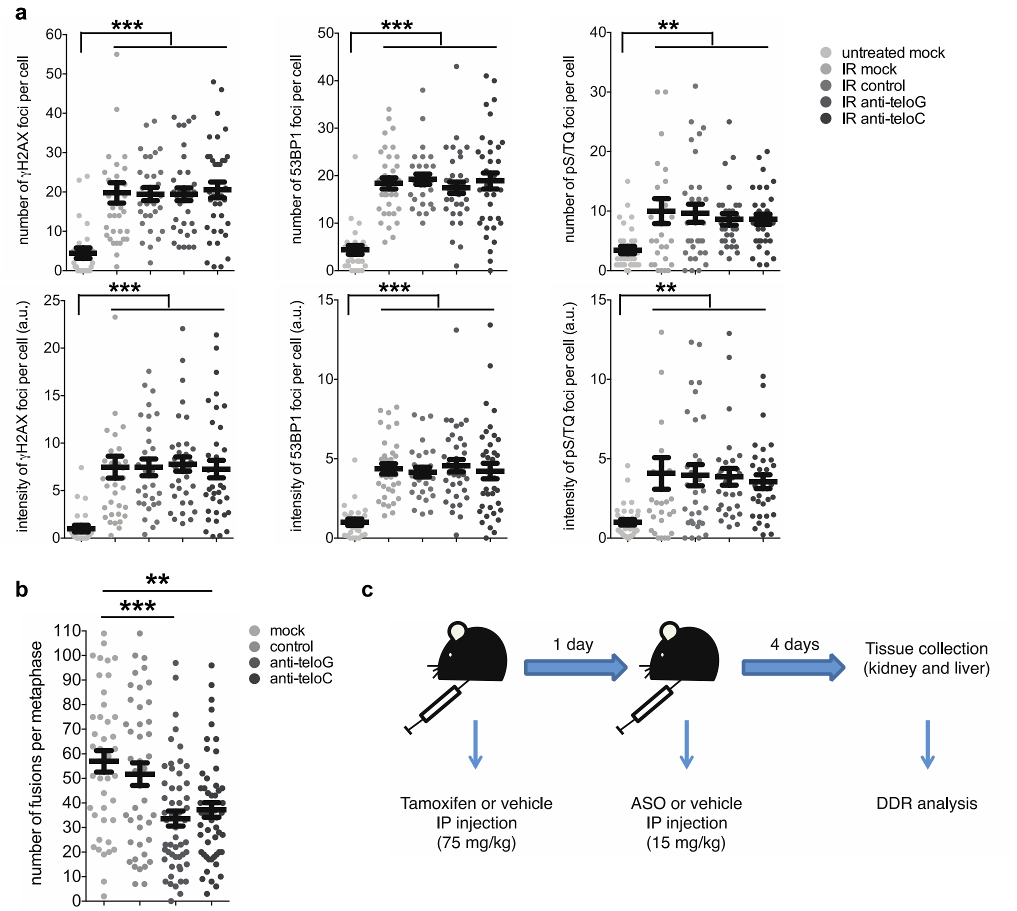

Supplementary Figure 6. (a) MEFs *Trf2*$^{F/F}$ were transfected with the indicated ASO. 48 hours later they were treated with ionizing radiation (IR, 1 Gy) and fixed after 1 hour. Dot plots show the number or intensity of DDR foci per cell (a.u. = arbitrary units). Lines depict the mean ± s.e.m. At least 30 cells per sample have been analysed; ** Pvalue < 0.01, *** Pvalue < 0.001, Student's t-test. (b) MEFs *Trf2*$^{F/F}$ were treated with 4-hydroxytamoxifen and transfected with the indicated ASO. 72 hours later metaphase spreads were stained with a telomeric PNA probe. Dot plots show the number of chromosomal fusions per metaphase. Lines depict the mean ± s.e.m. n = 3 independent experiments; at least 40 metaphases per sample have been analysed; ** Pvalue < 0.01, *** Pvalue < 0.001, Student's t-test. **(c)** Scheme of the ASO treatment in Trf2/p53/Rosa26 mice.

Supplementary Figure 7

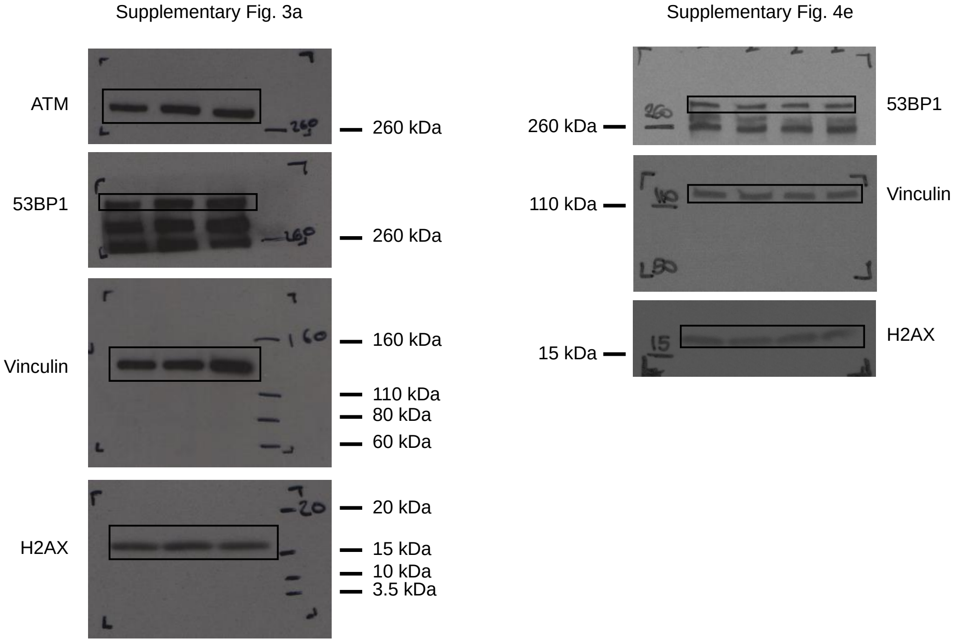

Supplementary Figure 7. Full scans.
